# Supplementary material for: MetaRibo-Seq measures translation in microbiomes
Source: Nat Commun. 2020 Jun 29;11:3268. doi: 10.1038/s41467-020-17081-z (PMC7324362; doi:10.1038/s41467-020-17081-z)
Supplement: Supplementary file 10 — Supplementary Data 7 [file 41467_2020_17081_MOESM10_ESM.zip › File2/Confidence_High_Taxonomy/346516_out.krona.html]

Javascript must be enabled to view this page.

members
magnitude
magnitudeUnassigned
count
unassigned
taxon
rank

346516\_out

5

superkingdom
10239
1

1
10501

SRS023914\_contig\_number\_26644
family

2
superkingdom
4

1224
phylum
3

3
68525
subphylum

3
28221
class

order
213462
3

3
family
213468

3
species

SRS013687\_contig\_number\_17451SRS098571\_contig\_number\_56164SRS1041145\_contig\_number\_contig-100\_97.164158
1961451

201174
phylum
1

1
1760
class

1
order
85009

1
85015
family

genus
117156
1


SRS051031\_contig\_number\_19884
117157
species
1
